# Early proteomic and metabolomic signatures in diabetes associated with progression to diabetic retinopathy over 1–2 years

**DOI:** 10.3389/fendo.2026.1842620

**Published:** 2026-06-10

**Authors:** Jing Zhao, Yitao Cao, Zhi Chai, Hanghang Xie, Dandan Gu, Yanchun Zhang, Dongling Niu, Hongli Liu, Ting Lei

**Affiliations:** 1 Clinical Laboratory Center, Department of Laboratory Medicine, Xi’an People’s Hospital (Xi’an Fourth Hospital), Xi’an, Shaanxi, China; 2 Key Laboratory of Resource Biology and Biotechnology in Western China, Ministry of Education, College of Life Sciences, Northwest University, Xi’an, Shaanxi, China; 3 School of Medicine, Northwest University, Xi’an, China; 4 Shaanxi Eye Hospital, Xi’an People’s Hospital (Xi’an Fourth Hospital), Xi’an, China

**Keywords:** diabetic retinopathy, metabolomics, multi-omics, proteomics, risk stratification

## Abstract

**Background:**

The progression from diabetes mellitus (DM) to diabetic retinopathy (DR) represents a dynamic pathological process in which vascular and metabolic alterations gradually lead to retinal damage. Understanding the molecular changes during this transition is critical for timely identification and intervention.

**Methods:**

We performed DIA-based proteomics and untargeted metabolomics on longitudinal paired plasma samples from 15 DM patients who developed DR during 1–2 years. Differential expression, pathway enrichment, and protein–metabolite correlation analyses were performed. Key proteins were validated by ELISA in an independent cohort of 22 DM patients without DR and 22 NPDR patients, and their predictive performance for DR progression was assessed using receiver operating characteristic (ROC) curve analysis.

**Results:**

Proteomic analysis identified 57 differentially expressed proteins enriched in energy metabolism, detoxification, and cellular defense responses. Metabolomic profiling revealed 168 differential metabolites, primarily involved in taurine and hypotaurine metabolism, neuroactive ligand–receptor interaction, and tyrosine metabolism. Integrated proteomic–metabolomic analysis revealed significant correlations between proteins and metabolites. Eight candidate proteins were validated by ELISA, including three previously reported in DR (Annexin A1 (ANXA1), Glutathione Peroxidase 1 (GPX1), Glutathione S-transferase Mu 1 (GSTM1)) and five newly identified candidates (Aldehyde Dehydrogenase 18 family member A1 (ALDH18A1), Galactose-1-Phosphate Uridylyltransferase (GALT), Proteoglycan 2 (PRG2), Ubiquitin-activating Enzyme E1 (UBA1), Glucagon (GCG)). ROC analysis demonstrated strong discriminative performance for these proteins, with area under the curve (AUC) values ranging from 0.853 to 0.945.

**Conclusions:**

Our study reveals coordinated alterations in plasma proteins and metabolites during the progression from DM to DR. Integrated multi-omics analysis identified five novel candidate biomarkers, which may serve as potential targets for early detection and risk stratification of DR.

## 1 Introduction

Diabetic Retinopathy (DR) is a common microvascular complication in diabetes mellitus (DM) which leads to vision impairment and blindness worldwide. With increasing incidence of diabetes, the number of individuals affected by DR is projected to reach 191 million by 2030 (1). As a progressive disorder, DR evolves from metabolic dysregulation to overt retinal pathology, suggesting that early molecular perturbations may drive the subsequent structural alterations (2). However, current fundus photography–based screening typically detects abnormalities only after substantial retinal injury has occurred, resulting in delayed diagnosis and missed opportunities for early intervention (3). Therefore, identifying individuals at high risk and elucidating changes that drive the transition from DM to DR are essential to prevent or delay disease onset.

Advancements in proteomics and metabolomics technologies have created a unique opportunity to shift disease assessment from structural evaluation toward molecular detection (4). These high-throughput platforms provide comprehensive and quantitative measurements of proteins and metabolites, enabling systematic interrogation of early biological changes (5). Prospective studies have demonstrated that elevated levels of DCXR and GSTA3 independently predict the onset of overt diabetes within one year (6). Similarly, metabolomic studies have shown that circulating amino acid- and lipid-related metabolites are already altered during the transition from normoglycemia or prediabetes to T2DM, suggesting that metabolic dysregulation begins before the development of overt diabetes (7, 8). These molecular changes may provide an early window for risk stratification and intervention. In parallel, prolonged hyperglycaemia induces “metabolic memory,” whereby prior glycaemic exposure leaves persistent molecular imprints that influence future disease progression (9, 10). This implies that the molecular signatures of disease progression capture not only current dysregulation but also the enduring impact of prior metabolic injury.

Although proteomic and metabolomic profiling has advanced rapidly, current evidence provides limited insight into the early transition from DM to DR (4). Most studies are cross-sectional, comparing established disease states and thus capturing only static molecular differences rather than the dynamic biological trajectory leading to retinal pathology (5, 11, 12). Longitudinal investigations are scarce and typically span extended follow-up periods, missing the critical early 1–3-year window when microvascular injury first develops (5). Furthermore, interpretation of existing findings is confounded by heterogeneous clinical variables—such as diabetes duration, glycaemic levels, pharmacological treatment, and comorbidities—which strongly influence circulating proteins and metabolites, obscuring signals that may be specific to DR onset.

Therefore, this study aimed to identify early plasma proteomic and metabolomic signatures associated with the progression from DM to DR over a 1–2 years period. Using a paired longitudinal design in which each participant serves as their own control, we minimize inter-individual variability and enhance the sensitivity to detect true progression-related molecular changes. We hypothesize that distinct pathways activated during this transition may serve as predictive biomarkers, informing earlier risk stratification and intervention.

## 2 Materials and methods

### 2.1 Study design and samples preparation

A total of 15 pairs of patients who exhibited rapid progression from DM to DR within 1–2 years were recruited from a multicenter, community-based cohort. Detailed information about the diagnostic criteria and participant eligibility has been reported previously (13). For each patient, blood samples were collected before and after DR onset using EDTA anticoagulant tubes, followed by centrifugation at 1600×g for 10 minutes at 4 °C to obtain plasma. The resulting supernatant was carefully aliquoted and immediately stored at −80 °C.

This paired cohort was subsequently subjected to integrated omics profiling to capture early molecular perturbations associated with DR development. Plasma proteomic changes were quantified using data-independent acquisition (DIA) mass spectrometry, while untargeted metabolomic profiling was performed using LC-MS/MS.

To validate candidate proteins identified in the longitudinal discovery analysis, targeted ELISA assays were conducted in an independent validation subset drawn from the same cohort. This cohort consisted of 22 individuals with diabetes mellitus (DM) and 22 patients diagnosed with early-stage non-proliferative diabetic retinopathy (NPDR), thereby enabling assessment of biomarker performance across the critical transition from diabetes to overt retinal involvement. All participants provided written informed consent, and the study was conducted in accordance with the Declaration of Helsinki. The protocol was approved by the Ethics Committee of Xi’an People’s Hospital (Xi’an Fourth Hospital) (20220066).

### 2.2 Plasma proteomics

A total of 100μg of protein from each sample was loaded into 10 kDa ultrafiltration tubes for digestion and peptide preparation. Mass spectrometry data were acquired using the Astral system in data-independent acquisition (DIA) mode.

DIA data were processed using MSstats, including system error correction and sample normalization. Data quality was assessed by evaluating intra-group coefficients of variation (CVs), principal component analysis (PCA), and quantitative correlations among samples. Differential protein expression was assessed using a predefined comparison design and a linear mixed-effects model. Proteins with a fold change >1.5 and P < 0.05 were considered significantly differentially expressed. Multiple database searches against UniProt, GenBank, and RefSeq were performed for protein annotation and identification.

### 2.3 Untargeted metabolomics

Serum samples were thawed at 4 °C and mixed with methanol–acetonitrile–water (4:2:1, v/v/v) containing an internal standard. After vortexing and protein precipitation at –20 °C for 2 hours, samples were centrifuged at 25,000 × g for 15 minutes at 4 °C. The supernatant was dried under vacuum and reconstituted in methanol–water (1:1, v/v) prior to analysis. A pooled quality control (QC) sample was prepared by combining equal aliquots from all study samples to monitor system stability and correct for batch effects.

Untargeted metabolomic profiling was conducted using a Q Exactive HF Orbitrap mass spectrometer (Thermo Fisher Scientific, USA) in both positive and negative ion modes. Full MS and data-dependent MS/MS spectra were acquired following standard instrument settings. Raw data were processed with Compound Discoverer and metaX. Probabilistic quotient normalization and QC-based LOESS correction were applied to minimize technical variation. Metabolites with a coefficient of variation >30% in QC samples were excluded from further analysis.

Processed data were used for multivariate statistical analyses, including principal component analysis (PCA) and partial least squares discriminant analysis (PLS-DA). Variables importance in projection (VIP) scores were extracted from the PLS-DA model to prioritize relevant metabolites. Metabolite identities were annotated by querying the Human Metabolome Database (HMDB) based on accurate mass and MS/MS spectra.

### 2.4 Integrated proteome–metabolome correlation analysis

Integrated correlation analysis of differentially expressed proteins and quantified plasma metabolites data was performed to investigate systemic molecular interactions. Pairwise Spearman’s rank correlation coefficients were calculated between all quantified plasma metabolites and proteins. For each metabolite–protein pair, the analysis included only samples with non-missing values for both molecules, and pairs with fewer than six valid observations across the cohort were excluded to ensure statistical robustness. To account for the multiple comparisons inherent in high-dimensional data, the obtained p-values were adjusted using the Benjamini-Hochberg false discovery rate (FDR) method. Metabolite–protein pairs with an FDR-adjusted p-value less than 0.05 were considered statistically significant.

### 2.5 Bioinformatic analysis

Gene Ontology (GO) and Kyoto Encyclopedia of Genes and Genomes (KEGG) enrichment analyses were performed using the clusterProfiler R package (v4.4.1) with a hypergeometric test. Multiple testing was corrected using the Benjamini–Hochberg false discovery rate (FDR), and terms with FDR-adjusted p-values < 0.05 were considered significantly enriched. Protein–protein interaction (PPI) networks were constructed via the STRING database, and hub proteins were identified based on node connectivity. Integrated metabolite–protein networks were visualized in Cytoscape to highlight modules of coordinated molecular changes.

### 2.6 ELISA validation

Protein concentrations were quantified in an independent cohort of patients with DM (n = 22) and early-stage NPDR (n = 22) using commercially available enzyme-linked immunosorbent assay (ELISA) kits obtained from Shanghai Enzyme-linked Biotechnology Co., Ltd. (Shanghai, China), including S100 calcium-binding protein P (S100P) (ml105599), protein disulfide isomerase family A member 3 (PDIA3) (ml104981), aldehyde dehydrogenase 18 family member A1 (ALDH18A1) (ml022718), glutathione peroxidase 1 (GPX1) (ml057611), annexin A1 (ANXA1) (ml038038), Charcot–Leyden crystal protein (CLC) (ml218465), ubiquitin-activating enzyme E1 (UBA1) (ml298892), glucagon (GCG) (ml105976), glutathione S-transferase mu 1 (GSTM1) (ml063427), proteoglycan 2 (PRG2) (ml246535), DExD-box helicase 21 (DDX21) (ml229643), and galactose-1-phosphate uridylyltransferase (GALT) (ml271197). All assays were performed according to the manufacturers’ instructions. Briefly, plasma samples and standards were added to the wells, followed by incubation with biotin-labeled detection antibodies and horseradish peroxidase (HRP)-conjugated secondary antibodies. After color development with substrate solution, the reaction was terminated using stop solution, and optical density was measured at 450 nm using a microplate reader. All samples were analyzed in duplicate, and the mean values were used for statistical analysis.

A standard curve was generated by plotting the mean optical density (OD) of the duplicate standard wells against their known concentrations. Linear regression was performed to obtain the curve equation. The coefficient of determination (R²) was required to be greater than 0.99 to ensure an acceptable fit. The concentration of each unknown sample was then calculated by interpolating its mean OD value into the linear regression equation. Any sample with an OD value exceeding the range of the standard curve was re-assayed at an appropriate higher dilution.

### 2.7 Statistical analysis

Continuous variables are presented as mean ± standard deviation (SD) and categorical variables as counts (percentages). Paired comparisons between DM and DR stages were performed using the paired Student’s t-test for normally distributed variables or the Wilcoxon signed-rank test for non-normally distributed variables. In the validation cohort, the discriminative ability of ELISA-quantified proteins to distinguish DM from DR was assessed using receiver operating characteristic (ROC) curve analysis. The area under the ROC curve (AUC) was calculated to evaluate predictive performance. All statistical analyses were conducted in R (version 3.6.0), with two-sided *P* values < 0.05 considered statistically significant.

## 3 Results

### 3.1 Clinical characteristics of patients

To investigate clinical features associated with early progression from DM to DR, 15 patients who developed DR within a 1–2 years follow-up period were identified. Key clinical parameters, including systolic and diastolic blood pressure, fasting blood glucose, glycated hemoglobin (HbA1c), hematological indices, lipid profiles, and renal function markers were compared between patients without DR (NDR) and those who progressed to DR. No statistically significant differences were observed for these variables between the two groups. Notably, diastolic blood pressure (DBP) tended to be lower in the DR group (*P* = 0.07), whereas serum uric acid (UA) levels were slightly higher (*P* = 0.08), although these trends did not reach significance (**Table 1**). These findings suggest that conventional clinical measures may not fully capture the early pathophysiological changes driving the transition from DM to DR.

**Table 1:** Changes in clinical characteristics during progression from DM to DR.

Characteristics	NDR	DR	*P* value
*N*	15	15	
Anthropometric factors			
SBP (mmHg)	144.60 (27.48)	137.73 (19.34)	0.28
DBP (mmHg)	82.07 (9.39)	76.67 (7.35)	0.07
Hematological Parameters			
WBC (10^9^/L)	5.98 (2.05)	5.77 (1.92)	0.43
Neu (10^9^/L)	3.99 (1.87)	3.64 (1.62)	0.15
Lym (10^9^/L)	1.54 (0.43)	1.64 (0.65)	0.68
RBC (10^12^/L)	4.38 (0.41)	4.44 (0.39)	0.55
Hb (g/L)	134.71 (12.76)	135.73 (13.33)	0.69
Hct (%)	40.79 (3.80)	41.43 (3.74)	0.88
Biochemical data			
TG (mmol/L)	4.23 (1.06)	4.20 (1.03)	0.77
TC (mmol/L)	1.82 (1.56)	1.56 (0.97)	0.63
HDL-C (mmol/L)	1.35 (0.32)	1.33 (0.34)	0.18
HbA1c (%)	7.80 (1.45)	7.60 (1.16)	0.29
BUN (mmol/L)	5.40 (1.05)	5.82 (2.01)	0.26
UA (umol/L)	267.43 (54.99)	294.45 (62.33)	0.08
Scr (umol/L)	67.21 (7.47)	64.95 (8.71)	0.12
eGFR	83.89 (12.69)	87.95 (11.28)	0.17

Data were presented as number (%) for categorial variables and mean (SD) for continuous variables. SBP, Systolic Blood Pressure; DBP, Diastolic Blood Pressure; WBC, White Blood Cell; Neu, Absolute Neutrophil Count; Lym, Absolute Lymphocyte Count; RBC, Red Blood Cell Count; Hb, Hemoglobin; Hct, Hematocrit; TG, Triglycerides; TC, Total Cholesterol; HDL-C, High Density Lipoprotein Cholesterol; BUN, Blood Urea Nitrogen; UA, Uric Acid; Scr, Serum Creatinine; eGFR, estimated glomerular filtration rate; NDR, Non-Diabetic Retinopathy; DR, Diabetic Retinopathy

### 3.2 Differential protein expression in the DR patients

**Figure 1:**
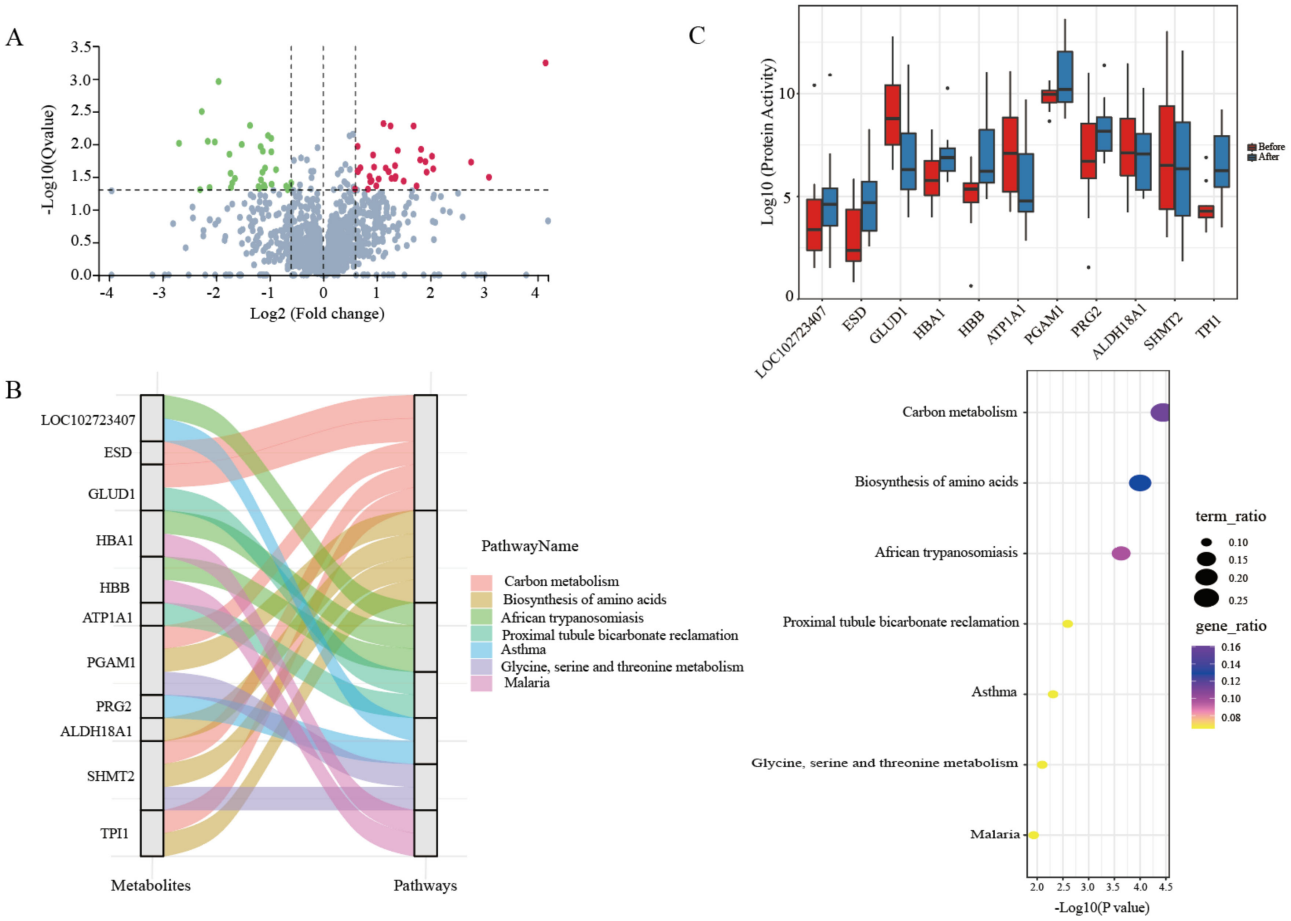
Identification and enrichment analysis of different expression proteins. **(A)** Volcano plot of DEPs. Significantly up-regulated and significantly down-regulated DEPs are indicated by red and green dots, respectively, while non-significantly differentially expressed are indicated by black dots. **(B)** KEGG pathway analysis on different proteins. **(C)** Different analysis of DR and NDR between the two groups.

A total of 16229 peptides and 1432 proteins were quantitatively identified from the plasma samples. Based on the thresholds of FC >1.5 and *P* < 0.05, 57 proteins were identified as differentially expressed, including 29 up-regulated and 28 down-regulated proteins (**Supplementary Table 1**). The volcano plot further illustrated the distinct proteomic landscape between the DR and NDR groups, demonstrating that individuals with rapidly progressing DR exhibit significant alterations in their plasma proteomic profiles (**Figure 1A**).

To better understand the interaction among the differentially expressed proteins, we then constructed protein-protein interaction (PPI) networks (**Supplementary Figure 1**). The analysis identified several high-degree nodes, including Triosephosphate isomerase 1 (TPI1) (degree = 11), Phosphoglycerate Mutase 1(PGAM1) (degree = 9), Chaperonin containing TCP1 subunit 7(CCT7) (degree= 8), Glutamate Dehydrogenase 1 (GLUD1) (degree = 7), ALDH18A1 (degree = 6), Glyoxalase 1 (GLO1) (degree = 6), PDIA3 (degree = 6), as well as Annexin A5 (ANXA5), DEAD-box Helicase 21(DDX21), Ribosomal Protein S3A (RPS3A), Serine Hydroxymethyltransferase 2 (SHMT2), Tyrosine 3-Monooxygenase/Tryptophan 5-Monooxygenase Activation Protein Beta (YWHAB) (degree = 5) and ANXA1 (degree = 4).

### 3.3 Enrichment analysis of differential expression proteins.

**Figure 2:**
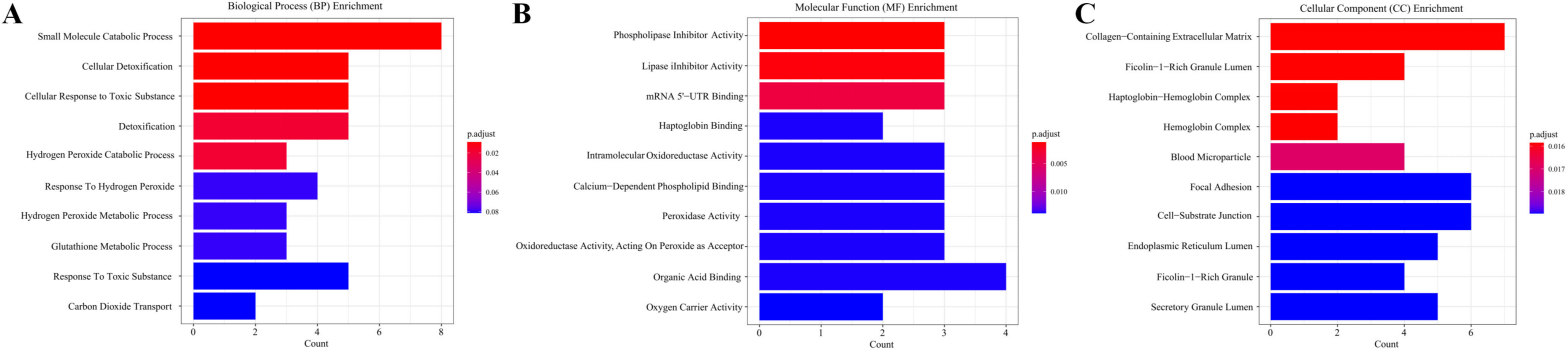
GO analysis of different expression proteins. **(A)** Biological process-based analyses of different expression proteins **(B)** Molecular function-based analyses of different expression proteins. **(C)** Cellular component-based analyses of different expression proteins.

To gain an insight into the biological significance of 57 regulated proteins, we conducted a GO analysis to explore the ontological functions of the differentially expressed proteins. The results revealed that these differentially expressed proteins were primarily involved in biological processes such as small-molecule catabolism, cellular defense and detoxification, and responses to toxic substances (**Figure 2A**). In terms of molecular function, the proteins were enriched in phospholipase and lipase inhibitor activity, as well as regulation of translation initiation (**Figure 2B**). The cellular component analysis indicated that the differential proteins were mainly localized to collagen-containing extracellular matrix structures, the luminal space of Ficolin-1–positive and other immune-related granules, and hemoglobin–haptoglobin complexes (**Figure 2C**).

KEGG pathway enrichment analysis was also performed to help determine the biological processes and functions of the differentially expressed protein. The significantly enriched pathways included carbon metabolism, amino acid biosynthesis, and African trypanosomiasis–related pathways (**Figure 1B**). Within these pathways, key proteins with altered expression included up-regulated Esterase D (ESD), HBA1, Hemoglobin Subunit Beta (HBB), Phosphoglycerate Mutase 1(PGAM1), Proteoglycan 2 (PRG2), and TPI1, as well as down-regulated Glutamate Dehydrogenase 1(GLUD1), ATP1A1, ALDH18A1, and SHMT2 (**Figure 1C**).

### 3.4 Analysis of metabolic profiles and metabolic pathways

**Figure 3:**
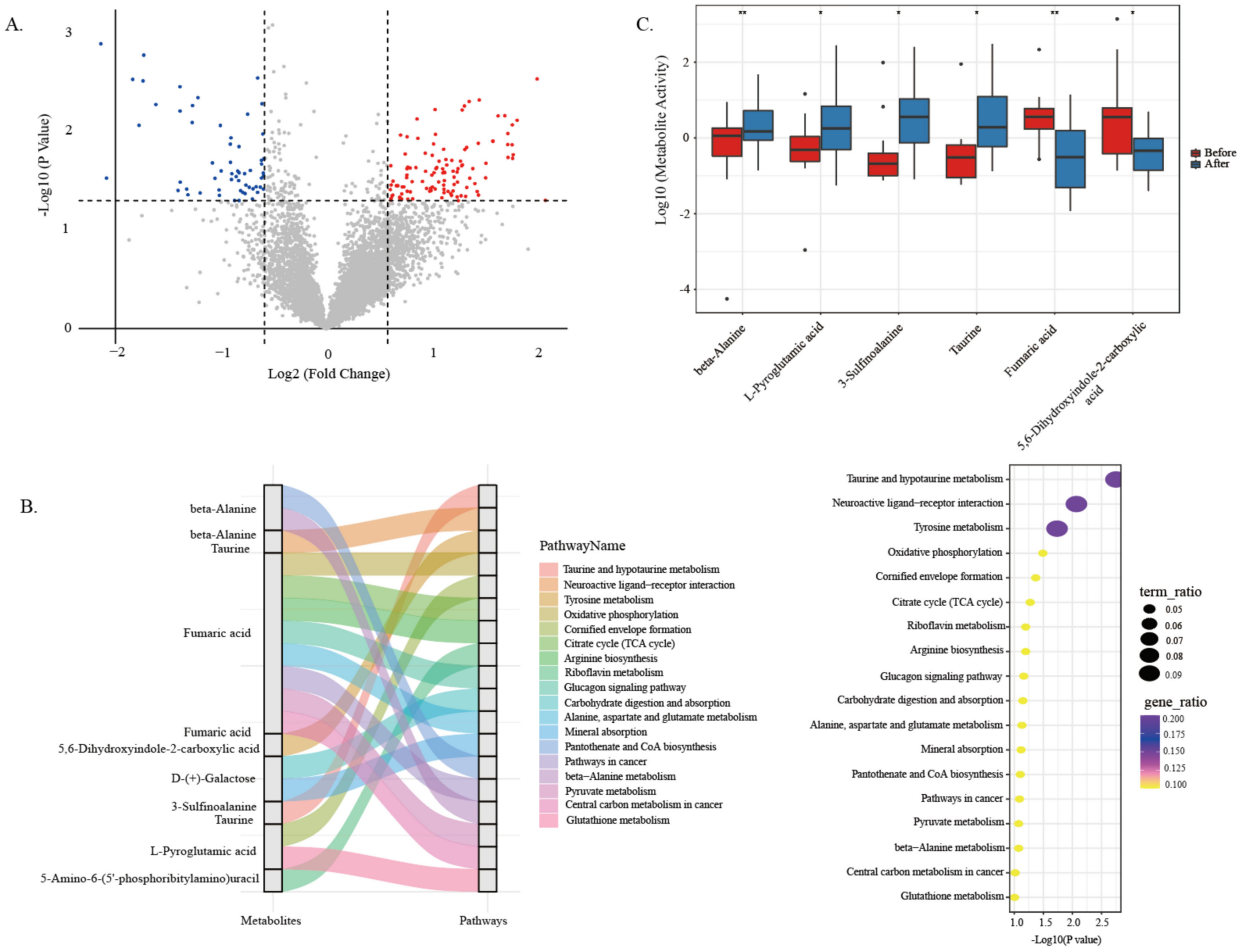
Identification and Enrichment Analysis of different metabolites. **(A)** Volcano plot of different metabolomics. Significantly up-regulated and significantly down-regulated metabolomics are indicated by red and green dots, respectively, while non-significantly differentially expressed are indicated by black dots. **(B)** KEGG pathway analysis on different metabolomics. **(C)** Different metabolomics analysis of DR and NDR between the two groups. : *P < 0.05, ** P<0.01.

To further understand the metabolic alterations in DR progression, we then performed untargeted metabolomics analyses to the plasma samples. Using the criteria of FC >1.5 & P <0.05 & VIP >1, a total of 168 differential metabolites were identified, including 110 up-regulated and 58 down-regulated metabolites (**Figure 3A**). Among them, 63 metabolites were annotated based on BMDM, mzCloud or HMDB databases (**Supplementary Table 2**).

Theses differential metabolites were predominantly enriched in several major chemical classes, including organic acids and derivatives, phenylpropanoids and polyketides, lipids and lipid-like molecules, and organic heterocyclic compounds, indicating broad metabolic dysregulation associated with DR. The most significantly down-regulated metabolomics were Fumaric acid. The most significantly up-regulated metabolomics were 3-Sulfinoalanine, Isoallolithocholic acid, Glutamyl pyruvate (**Figure 3B**).

Pathway enrichment analysis of these differential metabolites revealed significant involvement in taurine and hypotaurine metabolism, neuroactive ligand-receptor interaction and tyrosine metabolism. Within these pathways, key metabolites with altered expression included up-regulated B-alanine, L-pyroglutamic acid, 3-sulfoalanine, and taurine, as well as down-regulated fumaric acid and 5,6-dihydroxyindole-2-carboxylic acid (**Figure 3C**).

### 3.5 Integrated proteomic–metabolomic network analysis

**Figure 4:**
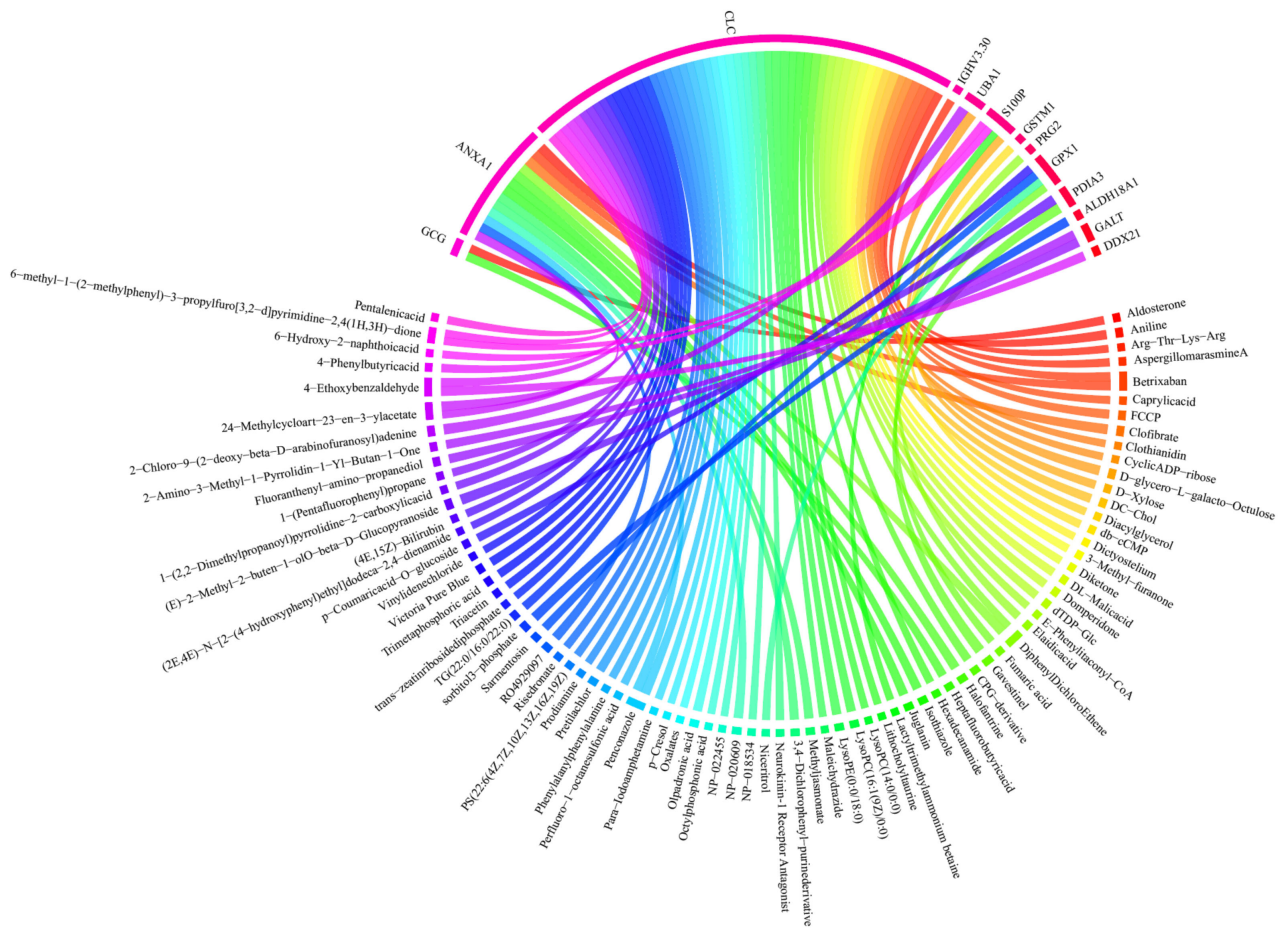
Correlation analysis between differentially expressed proteins and metabolites.

To elucidate the systemic interactions between proteomic and metabolomic changes during early DR progression, a correlation network integrating DEPs and metabolites was constructed (**Figure 4**), with detailed information provided in **Supplementary Table 3**. Notably, ALDH18A1 showed a positive correlation with sorbitol 3-phosphate, whereas GPX1 was negatively associated with niceritrol, fumaric acid, and sarmentosin. ANXA1 correlated with multiple metabolites, including aniline, betrixaban, clofibrate, and several lipid- and xenobiotic-related molecules. CLC exhibited the broadest connectivity, associating with 53 metabolites, including caprylic acid and DL-malic acid. Additional associations were observed for DDX21, S100P, UBA1, GCG, GSTM1, PDIA3, and PRG2. These findings suggest that the differentially expressed proteins are involved in extensive and diverse interactions with metabolites, reflecting coordinated proteomic–metabolomic changes that likely contribute to the early pathological processes underlying DR development.

### 3.6 Elisa validation

Based on the integrated proteomic–metabolomic correlation analysis, candidate proteins significantly associated with metabolites were selected for validation using ELISA in an independent cohort, including patients with DM (DM, n=22) and early-stage NPDR (NPDR, n=22). Protein concentrations were determined from standard curves generated using recombinant protein standards provided in the kits, based on OD values at 450 nm. The ELISA-based relative expression data for all 44 clinical samples are presented in **Supplementary Table 4**.

**Figure 5:**
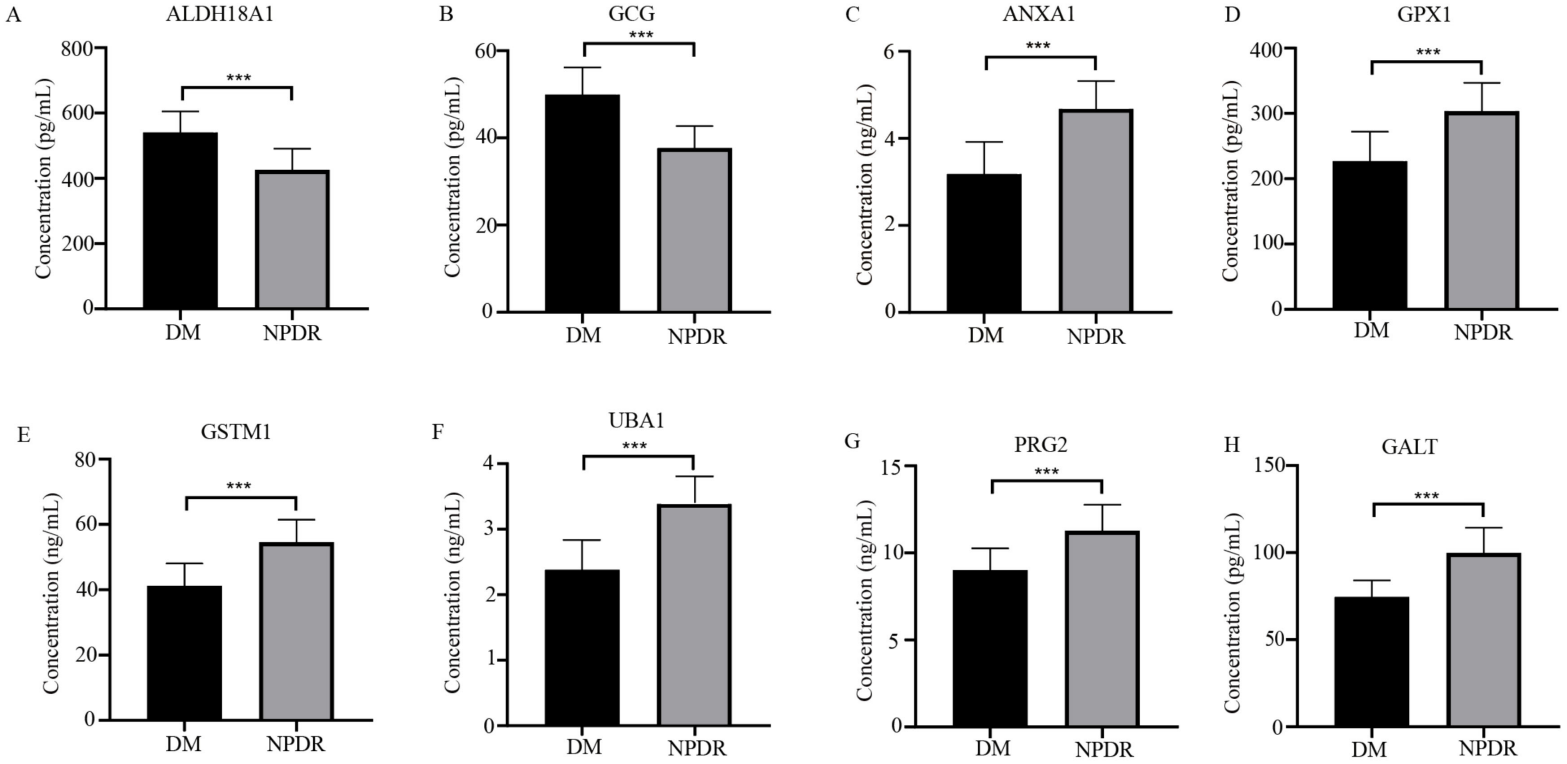
Plasma levels of eight candidate proteins in DM (n=22) and NPDR (n=22) patients. **(A)** Plasma concentrations of ALDH18A1. **(B)** Plasma concentrations of GCG. **(C)** Plasma concentrations of ANXA1. **(D)** Plasma concentrations of GPX1. **(E)** Plasma concentrations of GSTM1. **(F)** Plasma concentrations of UBA1. **(G)** Plasma concentrations of PRG2. **(H)** Plasma concentrations of GALT. Data are presented as mean ± standard deviation. Statistical significance was determined using the t-test; ***p < 0.001.

The validation results showed that ALDH18A1 and GCG were significantly decreased in NPDR compared with DM, whereas ANXA1, GPX1, GSTM1, UBA1, PRG2, and GALT were significantly elevated (**Figure 5**). These changes were consistent with those observed in the discovery proteomics dataset.

Interestingly, although CLC exhibited extensive correlations with multiple metabolites in the proteomics analysis, no significant difference was observed between the DM and NPDR groups in the ELISA validation. Furthermore, S100P, DDX21, and PDIA3 showed trends opposite to those observed in the discovery cohort (**Supplementary Figure 2**).

**Figure 6:**
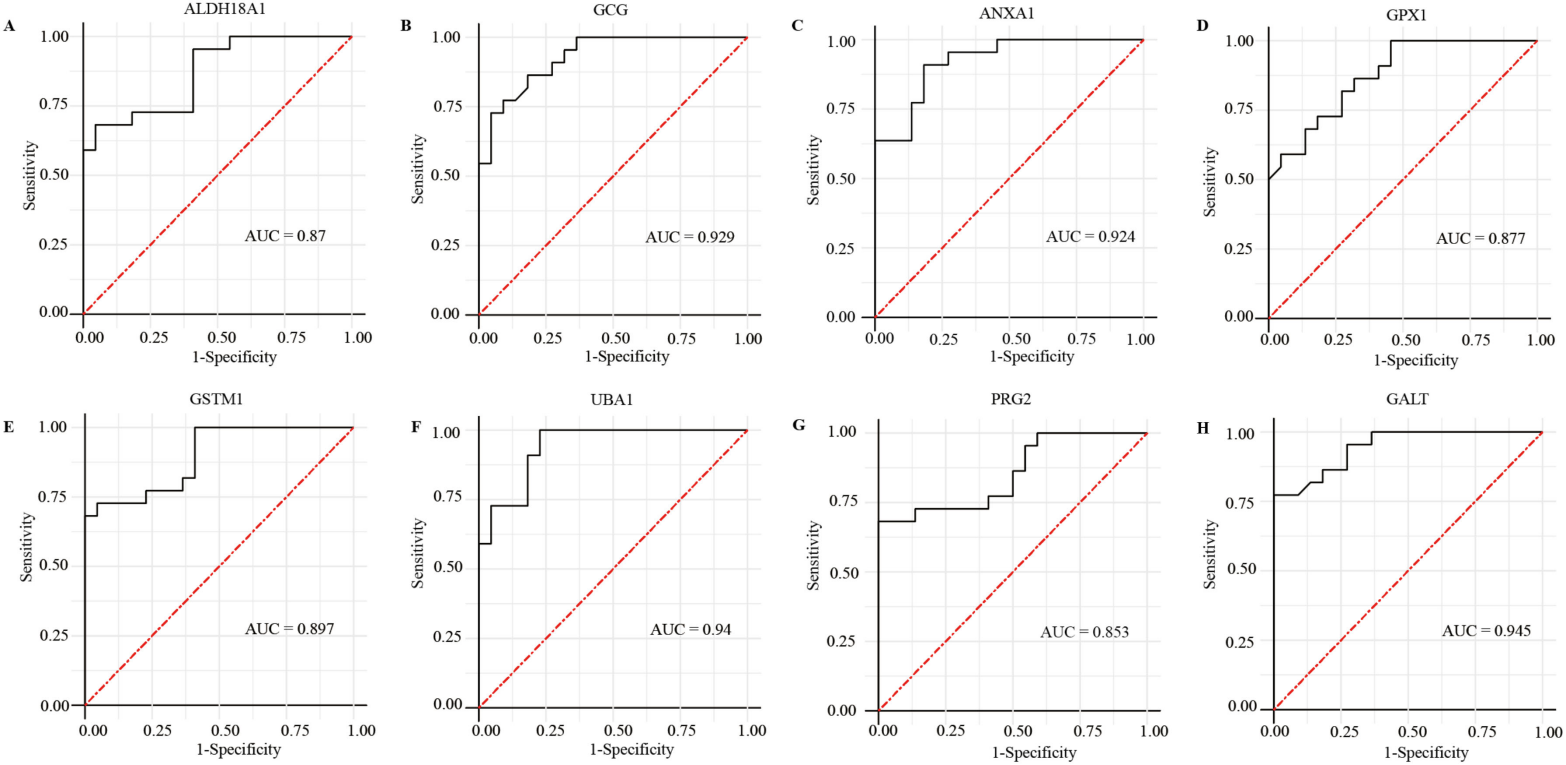
Receiver operating characteristic (ROC) curves of eight candidate plasma proteins for distinguishing DM and NPDR. **(A)** ROC curves for ALDH18A1; **(B)** ROC curves for GCG; **(C)** ROC curves for ANXA1; **(D)** ROC curves for GPX1; **(E)** ROC curves for GSTM1; **(F)** ROC curves for UBA1; **(G)** ROC curves for PRG2; **(H)** ROC curves for GALT.

Receiver operating characteristic (ROC) curve analysis demonstrated that several of these proteins exhibited good discriminatory performance, with area under the curve (AUC) values ranging from 0.853 to 0.945, highlighting their potential as early molecular indicators of DR progression (**Figure 6**).

## 4 Discussion

By leveraging paired plasma samples collected from the same individuals who exhibited rapid progression from DM to DR within 1–2 years, this study captured dynamic proteomic and metabolomic changes during a critical early window of disease development. This longitudinal design minimized inter-individual variability and enabled the identification of molecular signatures closely associated with DR initiation. Our study revealed significant alterations at both the protein and metabolite levels during the progression from DM to DR. Among the validated proteins, three ((ANXA1, GPX1, and GSTM1) were previously reported in DR, while five (ALDH18A1, GALT, PRG2, UBA1, and GCG) represent newly identified candidates. These findings provide insight into the molecular mechanisms underlying early DR progression and highlight potential targets for early detection and intervention.

### 4.1 Biological implications of newly identified proteins during DR progression

Among the newly identified candidates, ALDH18A1 exhibited a significant positive correlation with sorbitol-3-phosphate, a key intermediate of the polyol pathway activated under hyperglycemic conditions (14). ALDH18A1 encodes pyrroline-5-carboxylate synthase (P5CS), a mitochondrial enzyme involved in proline biosynthesis and closely associated with NADPH homeostasis, glutathione metabolism, mitochondrial function, and oxidative stress regulation (15). Previous studies have also reported dysregulated proline metabolism in diabetic and ischemic retinopathy, suggesting its involvement in retinal metabolic adaptation under pathological stress (16). Since hyperactivation of the polyol pathway consumes substantial amounts of NADPH and promotes ROS accumulation in diabetic retinopathy (17), the observed association between ALDH18A1 and sorbitol-3-phosphate may reflect coordinated metabolic remodeling and redox adaptation under chronic hyperglycemic stress rather than direct regulation of sorbitol metabolism. Consistent with this interpretation, our proteomic analysis also revealed increased expression of GSTM1, an important glutathione-dependent antioxidant enzyme involved in cellular detoxification and oxidative stress defense (18), suggesting activation of compensatory antioxidant responses during the transition from DM to NPDR.

Plasma GCG levels were significantly elevated in patients with DM and showed an inverse correlation with aldosterone levels. GCG encodes proglucagon-derived peptides, including glucagon and GLP-1, which are involved in glucose homeostasis and vascular regulation. Although antidiabetic medications may potentially influence with circulating GCG-related signaling, medication profiling showed that only one patient in the discovery cohort received SGLT2 inhibitor therapy during follow-up, with most participants treated with metformin and/or insulin (**Supplementary Table 5**). These findings suggest that the observed alterations in GCG levels were largely independent of medication-related confounding effects. Previous studies have shown that GLP-1 receptor signaling contributes to retinal vascular integrity and neuronal protection, while impaired GLP-1R expression is associated with increased retinal inflammation in DR (19). In addition, GLP-1 signaling has been reported to suppress aldosterone secretion and modulate renin–angiotensin–aldosterone system (RAAS) activity (20). Given the important role of aldosterone in retinal endothelial dysfunction and oxidative stress, the observed inverse association between GCG and aldosterone may reflect endocrine–vascular remodeling and potential cross-talk between incretin-related pathways and RAAS signaling during early DR progression.

UBA1 is the ubiquitin-activating E1 enzyme, the first step in the ubiquitin–proteasome system (UPS). The UPS is the principal mechanism for degrading damaged or misfolded proteins, and it is highly sensitive to metabolic stress. In diabetes, chronic hyperglycemia and oxidative stress impose heavy proteotoxic load, leading to UPS dysfunction (21). Similarly, PRG2 exhibited a negative correlation with elaidic acid, implicating early interactions between eosinophil granule proteins and lipid-mediated inflammatory pathways. GALT displayed inverse associations with galactose-derived metabolites, indicating dysregulation of alternative sugar metabolism, which may contribute to the accumulation of advanced glycation end-products and redox imbalance.

### 4.2 Reproducibility and validation of identified biomarkers

In addition to the newly identified candidate biomarkers, our study also identified several previously reported DR-related proteins, including ANXA1, GPX1 and GSTM1, further supporting the biological reliability of our findings. Previous studies have shown that these molecules are closely associated with inflammation, oxidative stress, and vascular dysfunction during DR progression (22). Among these proteins, we observed that ANXA1 was correlated with a large number of differential metabolites in our integrated analysis. Notably, several positively correlated metabolites were functionally related to PPARα activation, among which Clofibrate demonstrated the strongest biological relevance (23). Clofibrate is a classical PPARα agonist belonging to the fibrate family, and previous studies have demonstrated its protective effects in diabetic retinopathy, including reduction of retinal hard exudates, delayed progression of microvascular complications, and decreased need for laser treatment (24). Therefore, the strong positive correlation between Clofibrate and ANXA1 raises the possibility that PPARα-related signaling may partially exert retinal protective effects through ANXA1-associated pathways.

Similarly, GPX1 showed significant negative associations with fumarate-related metabolic alterations in the present study. As an important intermediate of the tricarboxylic acid cycle, fumarate accumulation has been linked to mitochondrial dysfunction and oxidative stress under diabetic conditions. GPX1 is a key glutathione-dependent antioxidant enzyme responsible for scavenging reactive oxygen species and maintaining mitochondrial redox homeostasis. Previous studies have shown that hyperglycemia-induced oxidative stress can suppress the mitochondrial glutathione–GPX axis and contribute to retinal injury in DR (25, 26). Therefore, the observed negative correlation may reflect a state in which fumarate accumulation and mitochondrial metabolic disturbance occur concomitantly with reduced GPX1-associated antioxidant capacity (27).

In addition, GSTM1 also exhibited a significant negative correlation with db-cCMP. GSTM1 is an important detoxification enzyme involved in glutathione metabolism and cellular redox regulation. Although the precise biological role of db-cCMP in DR remains unclear, cyclic nucleotide-related metabolites have been implicated in cellular stress signaling and metabolic regulation. The inverse association between GSTM1 and db-cCMP may indicate that disturbances in cyclic nucleotide metabolism are accompanied by impaired glutathione-dependent detoxification pathways under diabetic conditions.

Interestingly, although CLC exhibited extensive correlations with multiple metabolites in the correlation network analysis, its differential expression was not validated in subsequent analyses. Several factors may explain this discrepancy. First, the relatively limited sample size of the validation cohort may have reduced the statistical power to detect modest disease-associated differences. Second, technical differences between mass spectrometry-based proteomics and antibody-based ELISA assays may also contribute to inconsistent results. Proteomic approaches are generally more sensitive to subtle and context-dependent molecular alterations, whereas ELISA primarily reflects relatively stable circulating protein abundance. Previous studies have shown that CLC/Galectin-10 readily forms homodimers, protein complexes, and Charcot–Leyden crystal structures under inflammatory conditions (28). These structural and conformational changes may partially affect epitope accessibility in ELISA assays while remaining detectable by mass spectrometry-based approaches. Given its extensive associations with multiple differential metabolites, further validation in larger and independent cohorts is still required to clarify its precise biological and clinical significance.

### 4.3 Biological relevance of blood-derived biomarkers in reflecting retinal pathology

Although the biomarkers identified in the present study were derived from peripheral blood rather than ocular tissues, accumulating evidence suggests that circulating molecular alterations may partially reflect retinal pathological changes in DR. As a microvascular complication of diabetes, DR represents the ocular manifestation of systemic metabolic disturbance. Persistent oxidative stress, chronic inflammation, polyol pathway activation, and RAAS dysregulation not only alter circulating metabolic and proteomic profiles, but also contribute to retinal microvascular injury and blood–retinal barrier (BRB) dysfunction (29).

The BRB serves as a critical interface that separates the retina from the circulatory system, playing an essential role in preserving the homeostasis of the retinal microenvironment (30). As DR progresses, BRB integrity is progressively disrupted, leading to increased bidirectional exchange between the retina and the circulatory system. During this process, inflammatory cytokines, oxidative stress-related molecules, and aberrant metabolites generated in the local pathological retinal environment may enter the peripheral circulation (31).

Previous studies have identified partially shared differential metabolites and inflammatory molecules between the plasma and vitreous humor of DR patients, suggesting a degree of biological consistency between the peripheral circulation and the intraocular microenvironment (29, 32). Consequently, DR-related molecular changes are not confined to the local retinal tissue but may be concurrently reflected in the peripheral circulation. Given the complexity of DR pathophysiology, future studies integrating ocular tissues, animal models, or multi-tissue validation will be important to further determine the precise tissue origins of these circulating biomarkers.

### 4.4 Strengths and limitations

This study has serval strengths. First, the paired longitudinal design, which analyzed plasma samples collected from the same individuals before and after the onset of DR, thereby minimizing confounding effects related to genetic background, lifestyle, and environmental exposures. Second, by focusing on the earliest clinically detectable stage of DR, the study captures molecular alterations occurring at a critical transition point, providing direct insight into early pathogenic processes. Third, the integrated proteomic and metabolomic approach enables a multidimensional characterization of disease-related changes, with proteomics reflecting alterations in functional protein networks and metabolomics representing their integrated systemic consequences. Importantly, the validation of candidate protein biomarkers in an independent cohort enhances the robustness of the findings and supports their potential translational relevance for early risk assessment and biomarker development in DR.

Several limitations should also be consideration. First, although robust associations were identified, causal relationships between candidate proteins and DR progression remain to be established, Second, functional validation in retinal cell types and experimental models is required to delineate tissue-specific mechanisms. Third, although an independent validation cohort was included, the overall sample size remained relatively limited, which may reduce the statistical power and generalizability of the findings. Therefore, the predictive performance and clinical utility of the eight-protein panel still require further validation in larger prospective and multicenter cohorts.

In conclusion, our study reveals significant changes in both proteomic and metabolomic profiles during the progression from DM to DR. Importantly, we identified five novel circulating proteins with potential as early biomarkers. These biomarkers are linked to key processes such as metabolic stress, inflammation, and vascular dysfunction, offering valuable insights for early detection and intervention in DR. These findings not only provide candidate molecular targets for early risk stratification but also offer translational insights that could inform the development of preventive and therapeutic strategies for DR before clinically overt retinal damage occurs.

## Data Availability

The datasets presented in this study can be found in online repositories. The names of the repository/repositories and accession number(s) can be found in the article/**Supplementary Material**.

## Supplementary material

Supplementary Figure 1 The protein-protein interaction networks of the differentially abundant proteins.

Supplementary Figure 2 Comparison of validated protein expression between DM and NPDR participants.
