# Supplementary figures and images for: Early proteomic and metabolomic signatures in diabetes associated with progression to diabetic retinopathy over 1–2 years
Source: Front Endocrinol (Lausanne). 2026 Jun 10;17:1842620. doi: 10.3389/fendo.2026.1842620 (PMC13290453; doi:10.3389/fendo.2026.1842620)

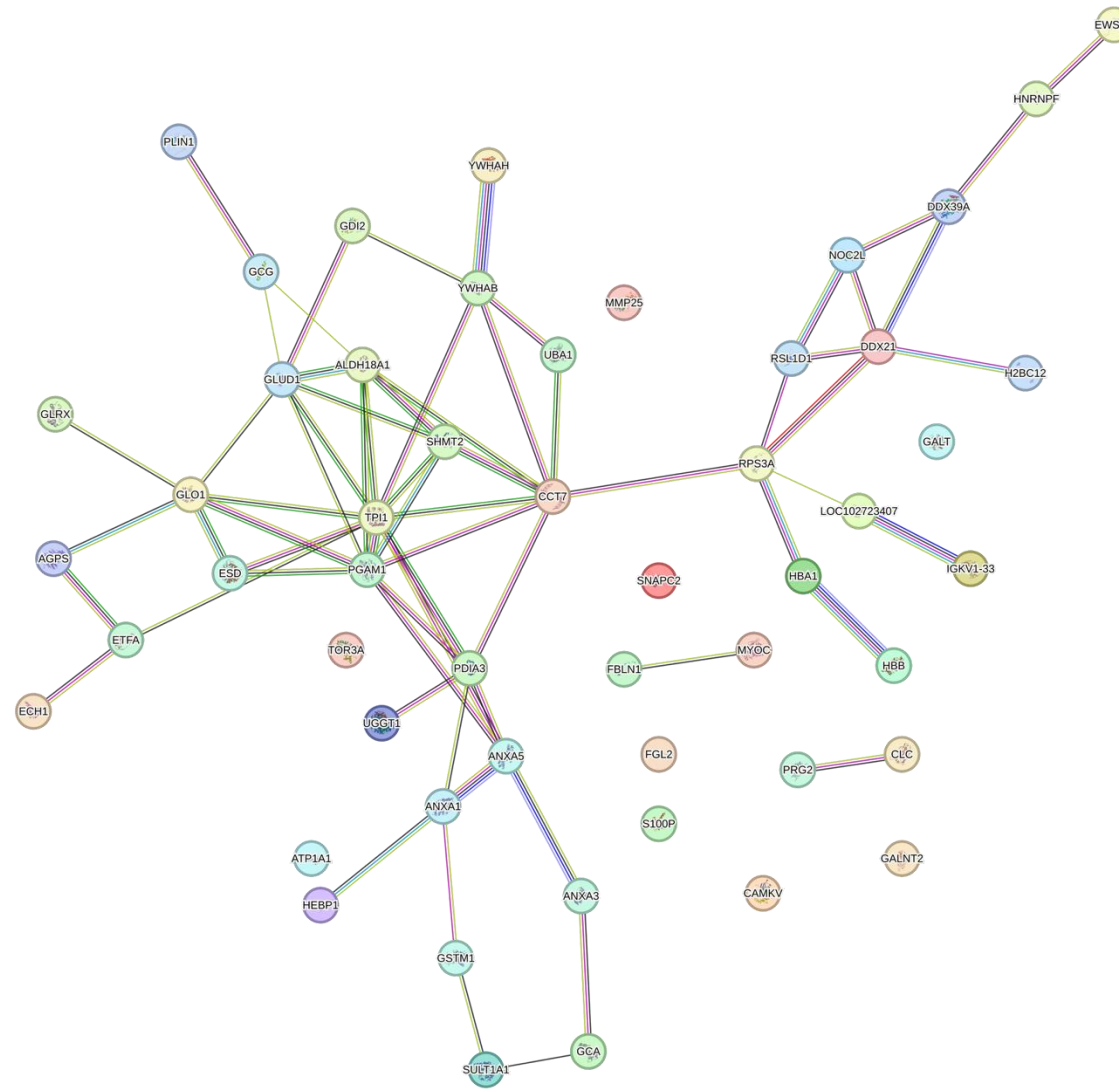

**Figure S1** The protein-protein interaction networks of the differentially abundant proteins

Supplement: Supplementary Figure 1 — The protein-protein interaction networks of the differentially abundant proteins. [file Image1.pdf]
