# Supplementary material for: Early proteomic and metabolomic signatures in diabetes associated with progression to diabetic retinopathy over 1–2 years
Source: Front Endocrinol (Lausanne). 2026 Jun 10;17:1842620. doi: 10.3389/fendo.2026.1842620 (PMC13290453; doi:10.3389/fendo.2026.1842620)
Supplement: Supplementary Figure 2 — Comparison of validated protein expression between DM and NPDR participants. [file Image2.pdf]

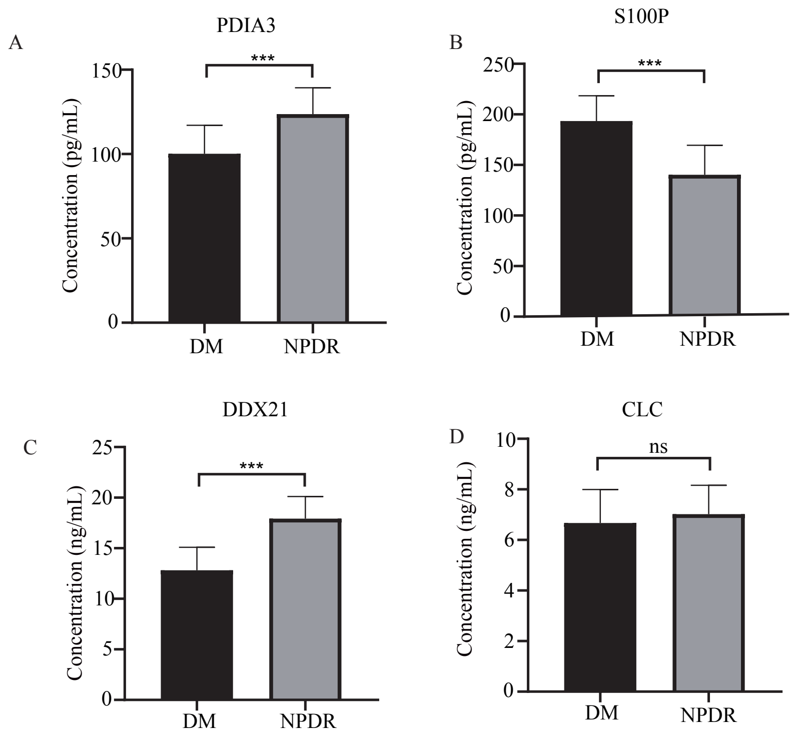

Figure S2 Comparison of validated protein expression between DM and NPDR participants. A. Plasma concentrations of PDIA3. B. Plasma concentrations of S100P. C. Plasma concentrations of DDX21. D. Plasma concentrations of CLC. ***p < 0.001; ns, not significant.
